# Analysis of plasmid genes by phylogenetic profiling and visualization of homology relationships using Blast2Network

**DOI:** 10.1186/1471-2105-9-551

**Published:** 2008-12-21

**Authors:** Matteo Brilli, Alessio Mengoni, Marco Fondi, Marco Bazzicalupo, Pietro Liò, Renato Fani

**Affiliations:** 1 Department of Evolutionary Biology, University of Florence, via Romana 17, I-50125 Florence, Italy; 2 Computer Laboratory, University of Cambridge, 15 JJ Thompson Avenue, Cambridge, CB3 0FD, UK; 3 Laboratoire de Biometrie et Biologie Evolutive, UMR CNRS 5558, Lyon, France

## Abstract

**Background:**

Phylogenetic methods are well-established bioinformatic tools for sequence analysis, allowing to describe the non-independencies of sequences because of their common ancestor. However, the evolutionary profiles of bacterial genes are often complicated by hidden paralogy and extensive and/or (multiple) horizontal gene transfer (HGT) events which make bifurcating trees often inappropriate. In this context, plasmid sequences are paradigms of network-like relationships characterizing the evolution of prokaryotes. Actually, they can be transferred among different organisms allowing the dissemination of novel functions, thus playing a pivotal role in prokaryotic evolution. However, the study of their evolutionary dynamics is complicated by the absence of universally shared genes, a prerequisite for phylogenetic analyses.

**Results:**

To overcome such limitations we developed a bioinformatic package, named Blast2Network (B2N), allowing the automatic phylogenetic profiling and the visualization of homology relationships in a large number of plasmid sequences. The software was applied to the study of 47 completely sequenced plasmids coming from *Escherichia*, *Salmonella *and *Shigella *spps.

**Conclusion:**

The tools implemented by B2N allow to describe and visualize in a new way some of the evolutionary features of plasmid molecules of Enterobacteriaceae; in particular it helped to shed some light on the complex history of *Escherichia*, *Salmonella *and *Shigella *plasmids and to focus on possible roles of unannotated proteins.

The proposed methodology is general enough to be used for comparative genomic analyses of bacteria.

## Background

Despite the huge amount of available sequences, few papers reported comparative analyses of entire plasmids with the aim of a complete classification of the functions they code for [1-4], and none considered all the sequences coming from entire genera or more inclusive taxonomic groups.

Nevertheless, plasmids are extremely important in microbial evolution, because they can be transferred between organisms, representing natural vectors for the transfer of genes and functions they code for [[5,6] and references therein]. In medical epidemiology and microbial ecology plasmids are thoroughly investigated because they often carry genes encoding adaptive traits such as antibiotic resistance, pathogenesis or the ability to exploit new environments or compounds [[7-9] and references therein].

While bacterial chromosomes show a relatively high conservation of their architecture, plasmid molecules are more variable concerning gene content and/or organization, even at short evolutionary distances. Indeed, plasmid genes can be considered to be under differential selection, while moving around the bacterial community. Moreover they have a dynamic structure, i.e. genes can be gained or lost from the plasmid molecule. Actually, the same plasmid can be hosted by different organisms inhabiting different environments (e.g.: pH, temperature and chemical composition) and cohabiting with different genetic backgrounds. These factors may shape both the functional role(s) of the proteins, and the compositional features of plasmid DNA, such as GC or oligomers contents, some of the last being a very specific signature even at close phylogenetic distances [10].

Despite their key role in the microbial world, at least two main issues concerning plasmids remain poorly investigated: i) the function of proteins they code for (see Additional file 1, more than 25% of proteins do not have assigned COG) and ii) the evolutionary dynamics of plasmids including their importance in bacterial evolution [11].

This latter point is often analyzed using phylogenetic methods that make use of rigorous statistical approaches to model the evolution of sequences (such as Maximum Likelihood or Bayesian inference). However, such methods are of restricted use in the case of plasmid molecules: they are computationally expensive when thousands of amino acid or nucleotide sequences are analyzed, and, moreover, require a set of homologous and universally shared sequences, that could be unavailable when studying plasmids.

To overcome these limitations we have developed a bioinformatic package (Blast2Network, B2N) having three main aims:

1) to reconstruct the evolutionary history of plasmids molecules by identifying those having the most similar gene content;

2) to assign a putative function to previously uncharacterized proteins. This task is fulfilled in two ways: by means of sequence similarity of unknown or hypothetical proteins to known ones and through a phylogenetic profiling approach. In this case the function of a protein is inferred by observing co-occurrence patterns. This is based on the idea that proteins involved in the same metabolic process or macromolecular complex tend to be maintained (or lost) together and that proteins which often occur together are likely to be functionally linked [12].

3) to provide an immediate visualization of the similarities existing among sequences. In fact, one of the outputs of the program is a network of sequence similarities in a format readable by the visualization software Visone .

To test the package, we focused the attention on plasmids harbored by members of the Enterobacteriaceae family of γ-Proteobacteria, which is one of the most studied divisions of bacteria and includes *Escherichia, Shigella*, and *Salmonella *genera, whose biomedical importance [13] has allowed to record a relatively high number of completely sequenced plasmids in a few species. Moreover, horizontal transfer of plasmids between them has been described [14], complicating the phylogenetic information on plasmids; lastly, several pathogenesis-associated phenotypes are plasmid-borne [15]. Consequently, the application of B2N to this dataset could allow to reveal the presence of relationships between known pathogenesis-associated proteins and those which have not been characterized yet.

## Methods

### Description of the program

**Figure 1:**
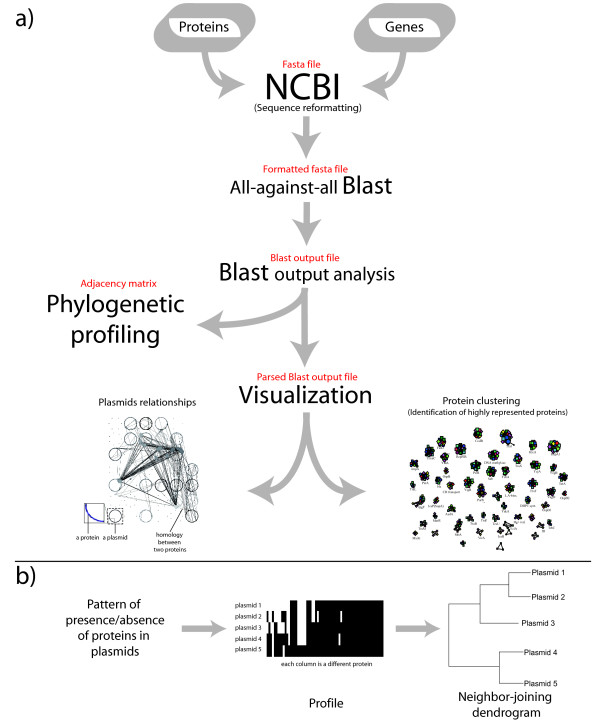
**B2N workflow and analysis**. a) Scheme of the data workflow in B2N. The visualization is realized using Visone software. The input of each module (i.e. the output of the previous one) is shown in red fonts. b) Phylogenetic profiling of molecules in the dataset. Using the matrix of occurrence patterns, groups of proteins are identified at different threshold values. A new matrix is obtained composed of a row for each plasmid in the dataset and a number of columns corresponding to the number of groups in the network. Each entry *i, j *of the matrix contains 1 if at least one protein from plasmid *i *is present in cluster *j*, 0 if no protein from plasmid *i *is present cluster *j*. This matrix is used for calculating distances using the Jaccard metric and dendrogram construction. This analysis identifies those plasmids that contain similar proteins. By applying the same workflow in the second dimension of the phylogenetic profiles matrix, it is possible to find those protein clusters having similar occurrence patterns.

The procedure implemented in B2N is schematically reported in Figure 1a, but several tasks can be performed separately because of the modular nature of our software. The main workflow starts from a file containing protein or nucleic acid sequences in standard NCBI fasta format. This is used as an input to gather information on source sequences from the NCBI website. Several files are automatically generated for reference along with the corresponding nucleotide sequences for both genes and source sequences (e.g. the genome or the plasmid encoding the proteins used as input). Input sequences are then screened one against each other using BLAST [16]. The resulting output is parsed in the form of an adjacency matrix that describes the global sequence similarities in the dataset where each entry *w*_*ij *_reflects the similarity existing between protein *i *and *j*. The user is initially prompted to choose two different selection criteria for alignments: an E-value threshold and an alignment length cut-off; after setting these parameters, all alignments passing the selection criteria are inserted in the matrix. Moreover, the user can specify the nature of the similarity score to be used, i.e. identity percentage or bit score; the bit score can also be normalized using the score of the alignment of the query with itself obtaining a value which is normalized on the alignment length. The weighted link values can be useful when comparing sequences from different species searching for those having the highest rate of horizontal transfer. This can be done in B2N specifying a distance matrix of house-keeping genes in Phylip format. The adjacency matrix obtained by parsing the BLAST output is the input for the phylogenetic profile method.

### Phylogenetic Profiling

This approach allows the analysis of co-occurrence patterns, metabolic reconstruction and so on. In details, by taking as input the adjacency matrix storing the sequence similarity values, B2N produces a rectangular matrix (as described in the central part of Figure 1b) composed by all the plasmids under analysis (rows) and all the protein clusters (columns) identified through a depth-first search of the adjacency matrix. Each position of the phylogenetic profile matrix will be "1" in the case a given plasmid (row) possesses (at least) one protein in the corresponding protein cluster (column), whereas it is filled with "0" in the opposite case.

One of the commonly used metrics for binary data comparison is the Jaccard similarity coefficient. Given two vectors of phylogenetic profiles in binary form (A and B in this case, with n observations), the Jaccard coefficient is defined as the size of the intersection divided by the size of the union of the sample sets: J(A, B) = |A ∩ B|/|A ∪ B|. The 'Jaccard distance', which measures dissimilarity between sample sets, is obtained by dividing the difference of the sizes of the union and the intersection of two sets by the size of the union: J_δ _(A, B) = |A ∪ B| - |A ∩ B|/|A ∪ B| = 1 - J(A, B).

The Jaccard coefficient is a useful measure of the overlap that the attributes of 'A' and 'B' share. Each attribute of 'A' and 'B' can either be 0 ('absence') or 1 ('presence'). The total number of each combination of attributes for both 'A' and 'B' are specified as follows: M_11 _(M_00_) represents the total number of attributes where 'A' and 'B' both have a value of 1 (0). M_01 _(M_10_) represents the total number of attributes where the attribute of 'A' is 0 (1) and the attribute of 'B' is 1 (0). Each attribute must fall into one of these four categories, meaning that their sum equals n. The Jaccard similarity coefficient is J = M_11_/(M_01_+ M_10_+ M_11_). Blast2Network calculates the Jaccard distance for both dimensions of the phylogenetic profiles matrix, which corresponds to the distance between plasmids in term of shared genes, and the distance between occurrence patterns of clusters in plasmids. The Jaccard distance matrices are then used for the construction of two neighbor-joining dendrograms (Figure 1b). The first one describes similarities in gene content of the plasmids, the other one groups together those protein clusters with the most similar occurrence pattern within plasmids. Random permutations of the original data allows to compute the statistical significance of the Jaccard distances.

### Network construction

B2N also outputs the BLAST post processing results as a network in Visone format , a freely available software for network visualization and analysis. In doing so, it takes advantage of several information: the position and the color of the nodes (proteins) in the network correspond to the plasmid source, whereas the links indicate the existence of a given degree of sequence similarity between nodes. To reduce the dimensionality of the networks it is possible to use the Jaccard distance matrices to construct two hypergraphs where each plasmid or protein cluster, respectively, are collapsed to single nodes connected by edges whose values reflect the significance of the Jaccard distance calculated (see below and in Additional file 2).

### Additional tools

B2N can include additional information in the network, assigning to each node a numerical (or binary) value which can be visualized in Visone as the size of the node; this node-associated value might be a compositional measure, such as the GC content and/or the codon adaptation index [17,18] of the corresponding gene. To this purpose, B2N has two methods but the user can input its own list of values as a text file. The first built-in method writes node values corresponding to the GC content of a sequence, while the other one implements the dinucleotide analysis derived from [10] and [19], obtaining a composition-based dissimilarity index of a gene sequence with respect to the source plasmid (or genome). Considering each possible dinucleotide, say *xy*, and a gene *s*, *ρ*_*xy*(*s*)_*= (f*_*xy*(*s*)_/*f*_*x*(*s*)_*f_*y*(*s*)_*)*. From this value the program obtains *δ*_(*s*,*g*)_ = 1/16 * Σ |ρ_*xy*(*s*) _- *ρ*_*xy*(*g*)_| over all 16 dinucleotides, that is a measure of the compositional bias of a given sequence *(s)* with respect to a reference sequence *(g)* i.e. the genome or the entire plasmid. The *δ *can be used to detect genes that have been recently transferred and have since then maintained the compositional properties of the original plasmid.

### Sequence data source and software availability

The dataset used in this work is composed by all the proteins encoded by the available completely sequenced plasmid sequences from *Escherichia, Shigella*, and *Salmonella *genera (Table 1). Complete plasmid sequences were downloaded from the NCBI ftp website .

**Table 1:** Plasmids analyzed

**Plasmid**	**Organism**	**Length (nt)**	**# ORF**	**Accession Number**
R721	*Escherichia coli*	75582	91	NC_002525

p9123	*Escherichia coli*	6222	8	NC_005324

pC15-1a	*Escherichia coli*	92353	100	NC_005327

pCol-let	*Escherichia coli*	5847	7	NC_002487

pAPEC-O2-R	*Escherichia coli*	101375	119	NC_006671

pColK-K235	*Escherichia coli*	8318	7	NC_006881

pRK2	*Escherichia coli*	5360	6	NC__005970

pECO29	*Escherichia coli*	3895	2	NC_001537

CloDF13	*Escherichia coli*	9957	8	NC_002119

pBHRK18	*Escherichia coli*	5721	4	NC_005568

pBHRK19	*Escherichia coli*	5721	4	NC_005569

pFL129	*Escherichia coli*	6464	4	NC_005923

pAPEC-O2-ColV	*Escherichia coli*	184501	209	NC_007675

pCoo	*Escherichia coli*	98396	94	NC_007635

pB171	*Escherichia coli*	68817	80	NC_002142

pO113	*Escherichia coli*	165548	155	NC_007365

pLG13	*Escherichia coli*	6293	7	NC_005019

pIGAL1	*Escherichia coli*	8145	3	NC_005248

p1658/97	*Escherichia coli*	125491	141	NC_004998

pKL1	*Escherichia coli *KL4	1549	1	NC_002145

pO157	*Escherichia coli *O157:H7 str. Sakai	92077	184	NC_002128

pOSAK1	*Escherichia coli *O157:H7 str. Sakai	3306	3	NC_002127

pSFD10	*Salmonella choleraesuis*	4091	6	NC_003079

pOU1113	*Salmonella enterica*	80156	89	NC_007208

pC	*Salmonella enterica *serovar Enteritidis	5269	4	NC_003457

pBERT	*Salmonella enterica *subsp.*enterica *serovar Berta	4656	9	NC_001848

pKDSC50	*Salmonella enterica *subsp. *enterica *serovar Choleraesuis	49503	48	NC_002638

cryptic_plasmid	*Salmonella enterica *subsp.*enterica *serovar Choleraesuis	6066	7	NC_005862

pSCV50	*Salmonella enterica *subsp. *enterica *serovar Choleraesuis	49558	51	NC_006855

pSC138	*Salmonella enterica *subsp.*enterica *serovar Choleraesuis	138742	170	NC_006856

pHCM1	*Salmonella enterica *subsp. *enterica *serovar Typhi str. CT18	218160	235	NC_003384

pHCM2	*Salmonella enterica *subsp.*enterica *serovar Typhi str. CT18	106516	105	NC_003385

pP	*Salmonella enteritidis*	4301	3	NC_003455

pK	*Salmonella enteritidis*	4245	3	NC_003456

pB	*Salmonella enteritidis*	1983	1	NC_005002

R27	*Salmonella typhi*	180461	207	NC_002305

pSC101	*Salmonella typhimurium*	9263	6	NC_002056

R64	*Salmonella typhimurium*	120826	135	NC_005014

pU302S	*Salmonella typhimurium*	3208	4	NC_006815

pU302L	*Salmonella typhimurium*	84514	103	NC_006816

pSLT	*Salmonella typhimurium*	93939	112	NC_003277

pSB4_227	*Shigella boydii *Sb227	126697	148	NC_007608

pSD1_197	*Shigella dysenteriae *Sd197	182726	223	NC_007607

pWR501	*Shigella flexneri*	221851	293	NC_002698

pCP301	*Shigella flexneri *2a str. 301	221618	261	NC_004851

ColJs	*Shigella sonnei*	5210	3	NC_002809

pSS_046	*Shigella sonnei *Ss046	214396	238	NC_007385

The table lists the plasmids used in this work, their accession numbers, their host organism, their length, and the number of proteins their genes code for.

The software B2N with the user's manual can be directly requested to the authors and is also available as Additional file (Additional file 3).

## Results and discussion

### Visual representation of sequence homology network

**Figure 2:**
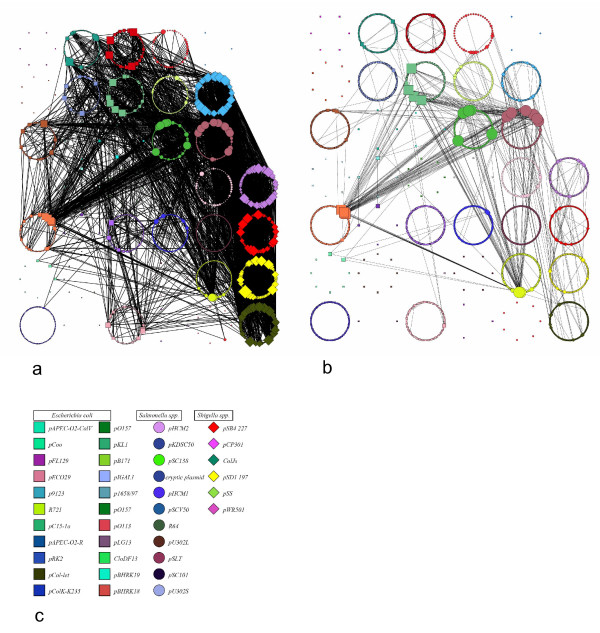
**Plasmid homology networks**. The output of B2N launched on the proteins encoded by 39 plasmids of three enterobacteria. Each protein in the dataset (see Table 1) is arranged circularly with proteins from the same source plasmid; proteins from the same plasmid are shown the same colour. Links connecting different nodes represent alignments found by BLAST (length > 70 amino acids and E-value<0.0001); consequently they describe the relationships existing between plasmids with a 90% (a) or 100% (b) identity cut-off; c) graphical legend. Symbols: squares, circles, and diamonds represent *E. coli*, *Salmonella *and *Shigella *plasmid proteins, respectively.

B2N was used to study the relationships existing between homologous proteins from all the completely sequenced plasmids available from three γ-Proteobacterial genera: *Escherichia*, *Shigella*, *Salmonella*. The dataset contains a total of 3701 ORFs, from 47 different plasmids (Table 1). To our knowledge, no attempt was made to describe in a meta analysis the overall body of plasmid sequence data in these species. Figure 2 shows the graphical representation of two networks generated with B2N using protein sequences in our dataset and using an aminoacid sequence identity threshold of 90% or 100% (Figure 2a and 2b respectively, where the thresholds are particularly high and the number of plasmids reduced to 39 out of 47 for clarity purposes). Proteins from the same plasmid are circularly arranged around the same centre and share the same color; proteins from the same genus are represented by the same shape (Figure 2c). The networks, obtained choosing an E-value threshold of 0.0001 and a minimum alignment length of 70 residues, have been visualized using the software Visone. The size of the nodes is proportional to the number of links they have. The analysis of Figure 2 revealed that most plasmids are strongly connected to others, but there are also plasmids exhibiting just few connections (see the section Phylogenetic profiling).

**Figure 3:**
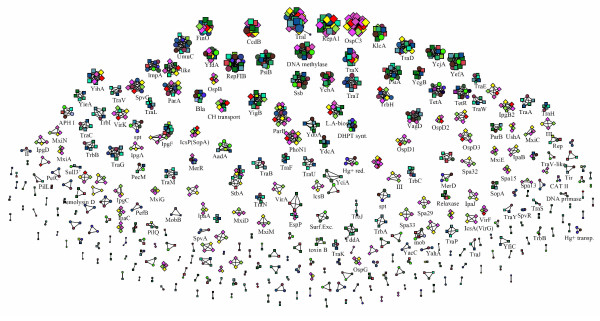
**Uniform visualization of protein clustering**. Uniform visualization of the similarity network for all of the 3701 proteins, displayed using a threshold identity for links of 40% (a degree of amino acid sequence identity sufficiently high to cluster together proteins that should perform the same function, and also allowing a better defined separation of all the main protein clusters [29,30]). Groups of homologous proteins are separated, allowing the identification of proteins that very likely share an identical/similar function. The labels for some groups of proteins discussed in the text or very common are shown: KlcA, antirestriction protein involved in the broad-host range of IncP plasmids; FinO, RNA chaperone related to repression of sex pilus formation; CcdB, protein involved in plasmid stability by killing bacteria that lose the plasmid during cell division; TetA and TetR, proteins responsible for resistance to tetracycline; Bla, β-lactamases; AadA, and DHPT synthase, proteins involved in resistance to aminoglycosides or sulfonamides, respectively. Tra and Trb, proteins requested for sex pilus formation; Mxi, Spa, Ipa, Ipg and Osp, proteins that are part of the type III secretion system.

Focusing on protein clusters instead of plasmids, we can arrange nodes in an uniform visualization, where nodes are clustered together if they directly or indirectly share at least one link (Figure 3, with a threshold of 40% identity). Quite interestingly, clustering of similar sequences at lower thresholds permits to assign a putative function to unknown or hypothetical proteins, and to discover the presence (if any) of functional classes or metabolic pathways that are more common in the network.

One of the problems faced with such complex data is the reduction of the dimensionality, so that important relationships can be more easily identified. Similarities in gene content between different plasmids can be better visualized by collapsing all the proteins belonging to the same plasmid in a single node. In this way a hypergraph is obtained where each node represents a single plasmid. The connection can be obtained from the plasmid vs plasmid Jaccard distance matrix or better, they can reflect the p-values matrix, so that each link in the hypergraph quantifies the significance of a given association between plasmids (showed in Additional file 2) and a simple hard thresholding allows changing the stringency for the inclusion of edges in the hypergraph.

### Network data analysis

The analysis of the network data represented in Figures 2 and 3 revealed several interesting features of the relationships among the sequenced plasmids of the three genera under investigation:

1) Out of a total of 3701 proteins in the dataset, we found 1633 (44%) and 2471 (66.7%) isolated nodes at a threshold of 90% or 100% of identity for links, respectively (Figure 2a and 2b).

2) Most plasmids contain at least some gene coding for highly interconnected proteins; however, some of them (e.g. pRK2, ColJs Cjl, pLG13, CloDF13) exhibited only few connections. Hence, these plasmids share few genes with the other members of the dataset at these threshold levels. This, in turn, may suggest that they might have experienced less recombination events than others.

3) Several proteins (about 40% of all the connected nodes) were found to be mobile elements (transposases, IS and transposons -related sequences), representing the most highly connected proteins in the network.

4) As shown in Figure 3, proteins shared by *Escherichia*, *Salmonella *and *Shigella *plasmids included: a) the antirestriction protein KlcA involved in the broad-host range of IncP plasmids [20]; b) the RNA chaperone FinO, related to repression of sex pilus formation [21,22]; c) the CcdB protein, which is involved in plasmid stability by killing bacteria that lose the plasmid [23].

5) Several clusters were composed by proteins shared by *Shigella *spp. and *Escherichia coli*; this finding is in agreement with the notion that they are considered to belong to the same species [24]. Moreover, several proteins were shared only by *E. coli *and *Salmonella *plasmids, including: the genetic determinants for antibiotic resistance such as TetA and TetR [25], β-lactamases (Bla) [25,27], genes for resistance to amino glycosides (AadA) and sulphonamides (DHPT synthase). A similar scenario was observed for sex pilus related proteins, such as Tra and Trb proteins: out of 22 different Tra groups, 21 contain proteins coming from *E. coli *and *Salmonella*, but 3 groups only (TraDI for DNA transport and TraX for pilin acetylation) have *Shigella *sequences. Likewise, out of 5 different Trb groups, we observed *Shigella *plasmid sequences in a single cluster (TrbH). Moreover, the proteins TraP, TrbA and TrbJ seem to be only present in plasmids from *E. coli*, while all the other sex pilus related proteins are shared with *Salmonella*. These data are in agreement with evidences for recent transfer of plasmid genes between enteroinvasive *Escherichia *and *Salmonella *[26,27].

Concerning the pathogenesis-related genes, *Shigella *plasmids seem to have a specific set of these genes, comprising at least some of the proteins of the type III secretion system (TTS), e.g.: Mxi, Spa, Ipa, Ipg and Osp proteins.

Finally, on the overall observation it appeared that besides the closer phylogenetic relationships existing between *E. coli *and *Shigella*, plasmid content appeared more similar among *E. coli *and *Salmonella *for what is concerned with antibiotic resistance and sex pilus formation.

### Phylogenetic Profiling

**Figure 4:**
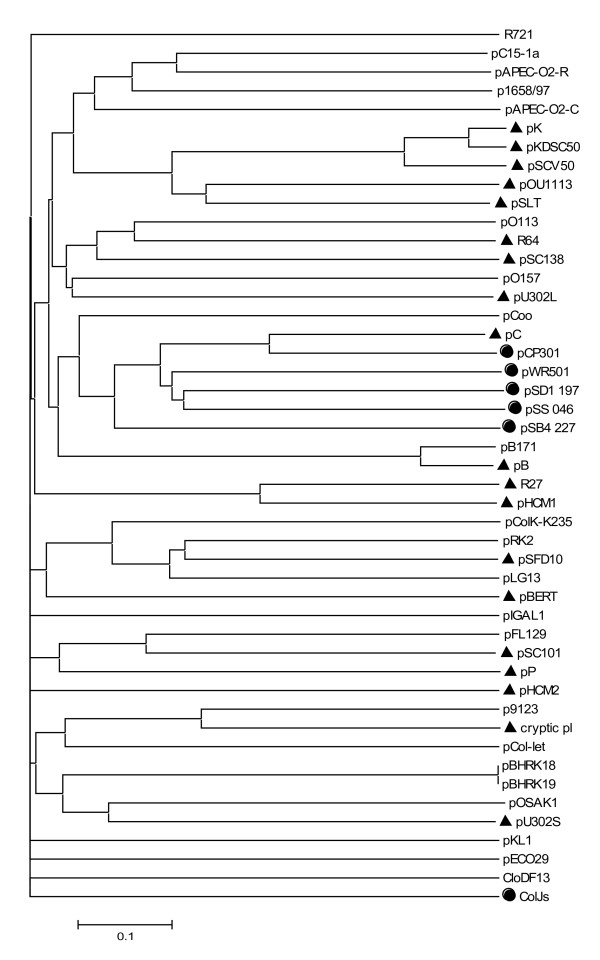
**Neighbor-Joining dendrogram of the plasmids from phylogenetic profiling**. Clustering of similarities in gene content of the plasmids obtained from their phylogenetic profile is reported (see text for details). Black circles or triangles before plasmid name refer to *Shigella *spp. or *Salmonella *spp. plasmids, respectively; *Escherichia coli *plasmids are not labeled.

**Figure 5:**
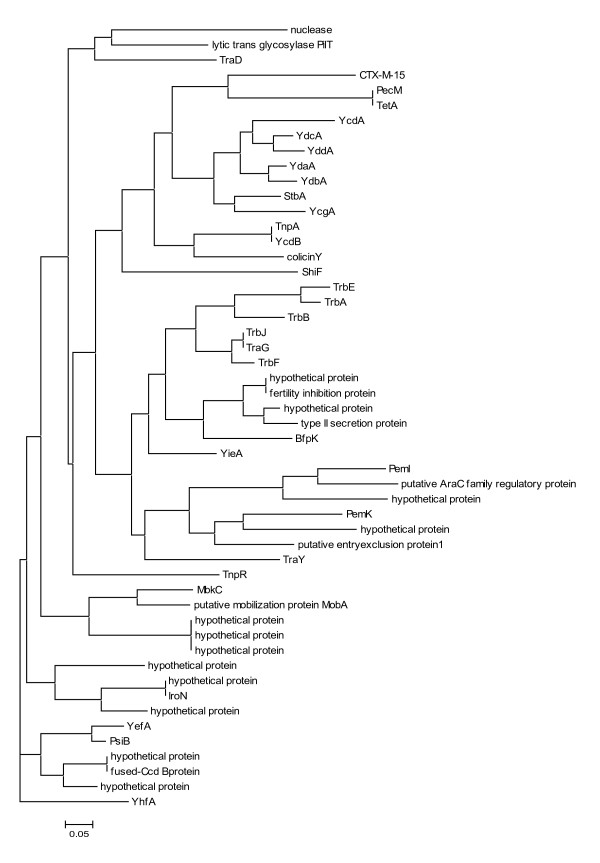
**Neighbor-Joining dendrograms of protein co-occurrence pattern from phylogenetic profiling**. Each cluster of this dendrogram includes those proteins that are commonly found together in the plasmids of the dataset reported in Table 1. Each hypothetical protein is associated with the GI of one representative of its corresponding protein cluster.

Data discussed in the previous paragraphs, that is which proteins join a given cluster, were stored by B2N into a text file, which represent the phylogenetic profile of the dataset used; this can be further used by the program to calculate two matrices storing the distances between profiles in the two dimensions (i.e. for plasmids and for proteins), as described in Methods. The corresponding neighbor-joining dendrograms, that describe the similarity in gene content of the plasmids and protein co-occurrence patterns are shown in Figure 4, Figure 5 and Additional file 4. Data reported in Figure 4 revealed that most of plasmids does not form tight clusters coherent with the taxonomic status of their respective host species (*E. coli*, *Salmonella *or *Shigella*). This finding suggests a complex evolutionary history of such plasmid replicons with massive horizontal transfer and gene rearrangements. In particular, plasmid pSFD10 from *Salmonella *grouped with two *E. coli *plasmids (pRK2 and pLG13).

A relevant exception is represented by five *Shigella *plasmids (pCP301, pSB4 227, pSD1 197, pWR501, and pSS 046) that form a unique clade (which, however, also includes pC plasmid from *Salmonella enterica*).

Figure 5 and Additional file 3 report the co-occurrence clustering for the protein dataset of the selected plasmids. In general, plasmids are believed to share very few common functions (mainly related to their replication and mobility), several accessory genes and a complex history of recombination events among either them or the host chromosome(s) [28]. Here, we actually show that most of the co-occurrence clusters are due to protein related to plasmid transfer (e.g. Trb and Tra proteins). Nevertheless, several clusters are present showing the co-occurrence of hypothetical proteins with proteins with predicted functions such as type II secretion proteins and pilins (BfpK), or with proteins involved in mobilization (MobA, MbkC) and virulence factors (IroN). These analyses may help in addressing experimental analyses for elucidating the functional role of these proteins.

## Conclusion

In conclusion, we report that the tools implemented by B2N allow to describe and to visualize in a new way some of the evolutionary features of plasmid molecules of Enterobacteriaceae; the most important results obtained by B2N on the Enterobacteriaceae dataset are related to the possibility, by means of phylogenetic profiling and network relationships of proteins, to uncover some of the molecular history, which shaped the evolution of this group of plasmids. In particular, data obtained suggested a large amount of horizontal transfer and rearrangement of plasmid molecules between *E. coli*, *Salmonella *and *Shigella*. Moreover, interestingly some plasmids from *Shigella *share a common history with *Salmonella *and several hypothetical proteins form co-occurrence clusters, suggesting possible roles in plasmid maintenance and/or pathogenesis, which could be investigated by conventional genetic techniques.

The proposed method is general enough to be proposed as a new tool for comparative genomic analyses of bacteria and can work at least within the range of phylogenetic distances enabling Blast to find homologs. For this reason, the B2N approach could help solving some questions linked to the presence of (few) well conserved functions within plasmid datasets from wide taxonomic ranges (e.g. functions related to transfer or replication). Moreover, possible applications of the method could include also chromosomal replicons, trying to depict histories of gene rearrangement and integration from plasmid to chromosomes and *viceversa*.

## Abbreviations

B2N: Blast2Network; TTS: type III secretion system.

## Authors' contributions

MBr participated in conceiving the idea, wrote the program and performed part of the analyses. AM, PL and RF participated in conceiving the idea. MF performed part of the analyses. MBa participated in discussing results. All authors contributed to draft the paper. All authors read and approved the final manuscript.

## Supplementary Material

Additional file 1 **Figure 1S – The functional activity of proteins from plasmid molecules present in GenBank database (as on March 2008)**. Histogram showing the putative roles of all the proteins (73909) encoded by the plasmids present in the NCBI repository. Each of the 73909 proteins was probed against the COG database  and its function was inferred according to the one assigned to the first BLAST hit of COG database. Data show that about 45% of the plasmid proteins deposited in the NCBI plasmids database have only a "general function" assignment or do not have any functional assignment at all. Click here for file

Additional file 2 **Figure 2S – Hypergraph of plasmid sequences**. Similarity network showing the relationships existing among plasmids listed in Table 1, shown at two distinct thresholds. Differently from Figure 2, now each node represents a single plasmid and links the overall protein content shared among entire plasmids. In details, the size of nodes is proportional to the number of links possessed by a given plasmid, whereas the thickness of links was computed using p-values of the Jaccard distance calculated in phylogenetic profiling analysis (see text), hence accounting for an overall estimation of the shared proteins by each plasmids in respect to the others. Click here for file

Additional file 3 **Software availability, requirements and user manual**. Project name: Blast2Network. Project home page: . Operating system(s): Platform independent. Programming language: Java. Licence: GNU GPL. Any restrictions to use by non-academics: no restriction. Click here for file

Additional file 4 **Figure 3S – Phylogenetic profile with GI numbers of represented proteins as in Figure **5. Protein co-occurrence patterns (see text for details) including the GI numbers of those proteins taken as representatives of each single cluster of Figure 3. Click here for file
