# Supplementary figures and images for: Analysis of plasmid genes by phylogenetic profiling and visualization of homology relationships using Blast2Network
Source: BMC Bioinformatics. 2008 Dec 21;9:551. doi: 10.1186/1471-2105-9-551 (PMC2640388; doi:10.1186/1471-2105-9-551)

Figure S1

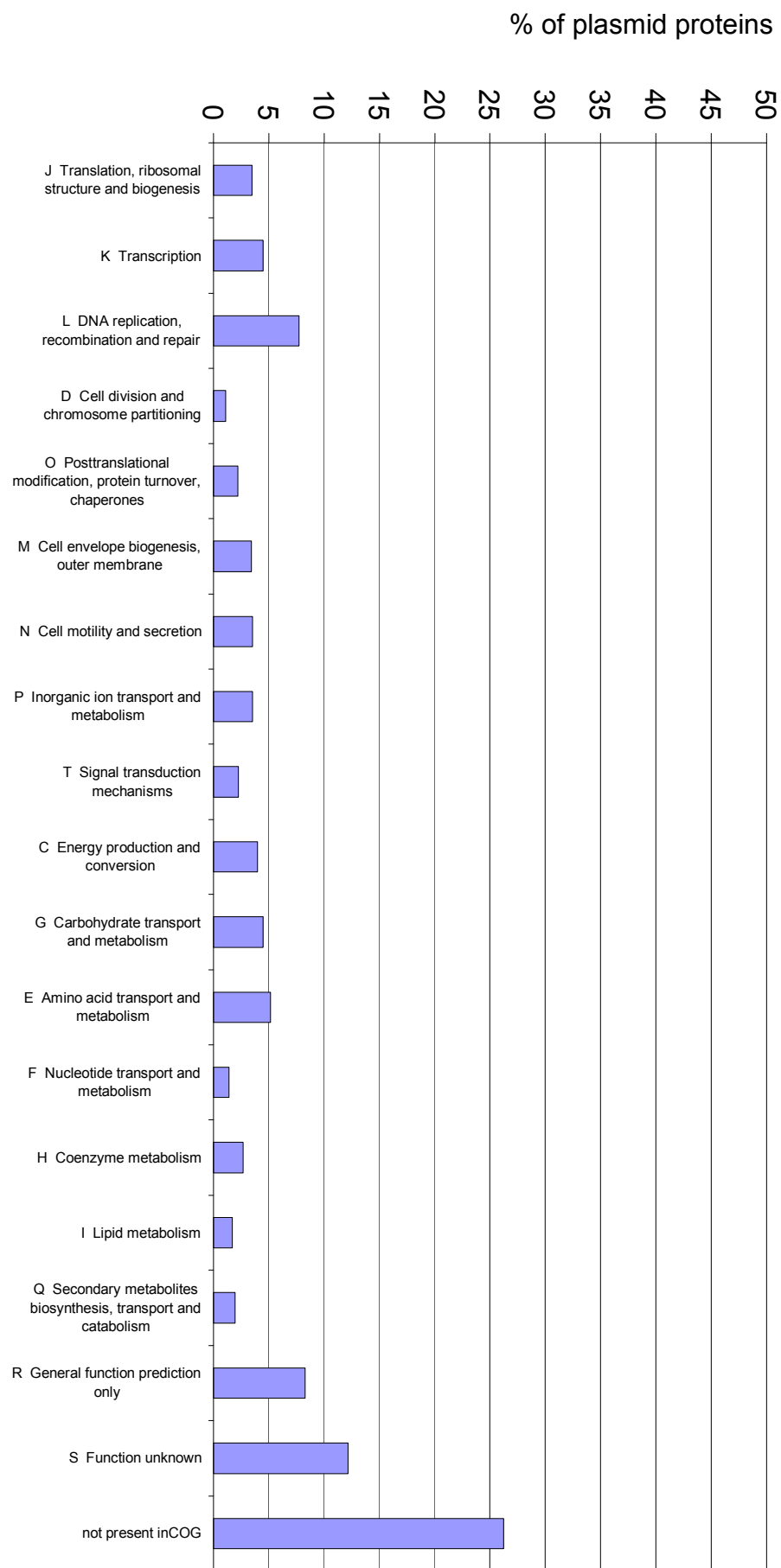

Supplement: Additional file 1 — Figure 1S – The functional activity of proteins from plasmid molecules present in GenBank database (as on March 2008). Histogram showing the putative roles of all the proteins (73909) encoded by the plasmids present in the NCBI repository. Each of the 73909 proteins was probed against the COG database and its function was inferred according to the one assigned to the first BLAST hit of COG database. Data show that about 45% of the plasmid proteins deposited in the NCBI plasmids database have only a "general function" assignment or do not have any functional assignment at all. [file 1471-2105-9-551-S1.pdf]

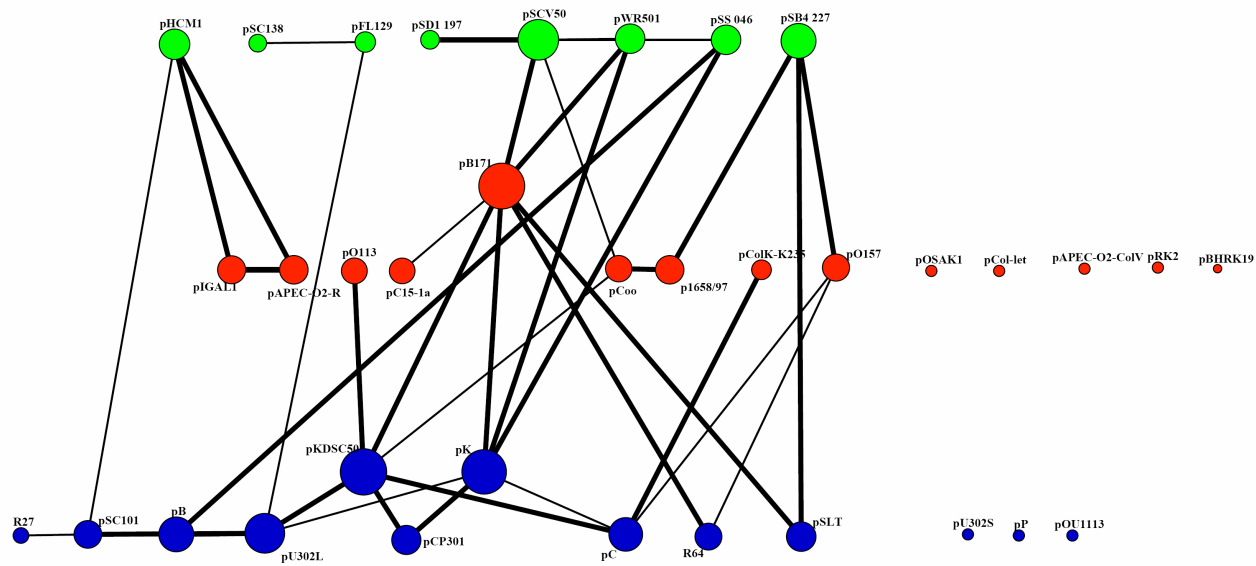

*Escherichia* plasmids

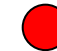

*Salmonella* plasmids

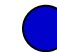

*Shigella* plasmids

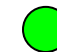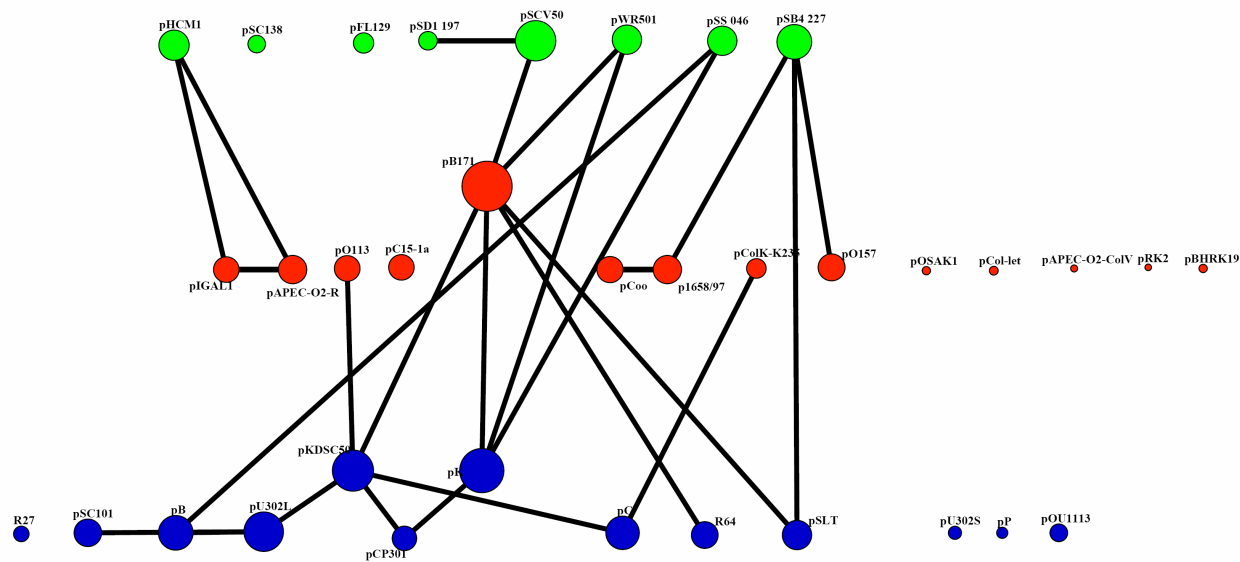

Supplement: Additional file 2 — Figure 2S – Hypergraph of plasmid sequences. Similarity network showing the relationships existing among plasmids listed in Table 1, shown at two distinct thresholds. Differently from Figure 2, now each node represents a single plasmid and links the overall protein content shared among entire plasmids. In details, the size of nodes is proportional to the number of links possessed by a given plasmid, whereas the thickness of links was computed using p-values of the Jaccard distance calculated in phylogenetic profiling analysis (see text), hence accounting for an overall estimation of the shared proteins by each plasmids in respect to the others. [file 1471-2105-9-551-S2.pdf]

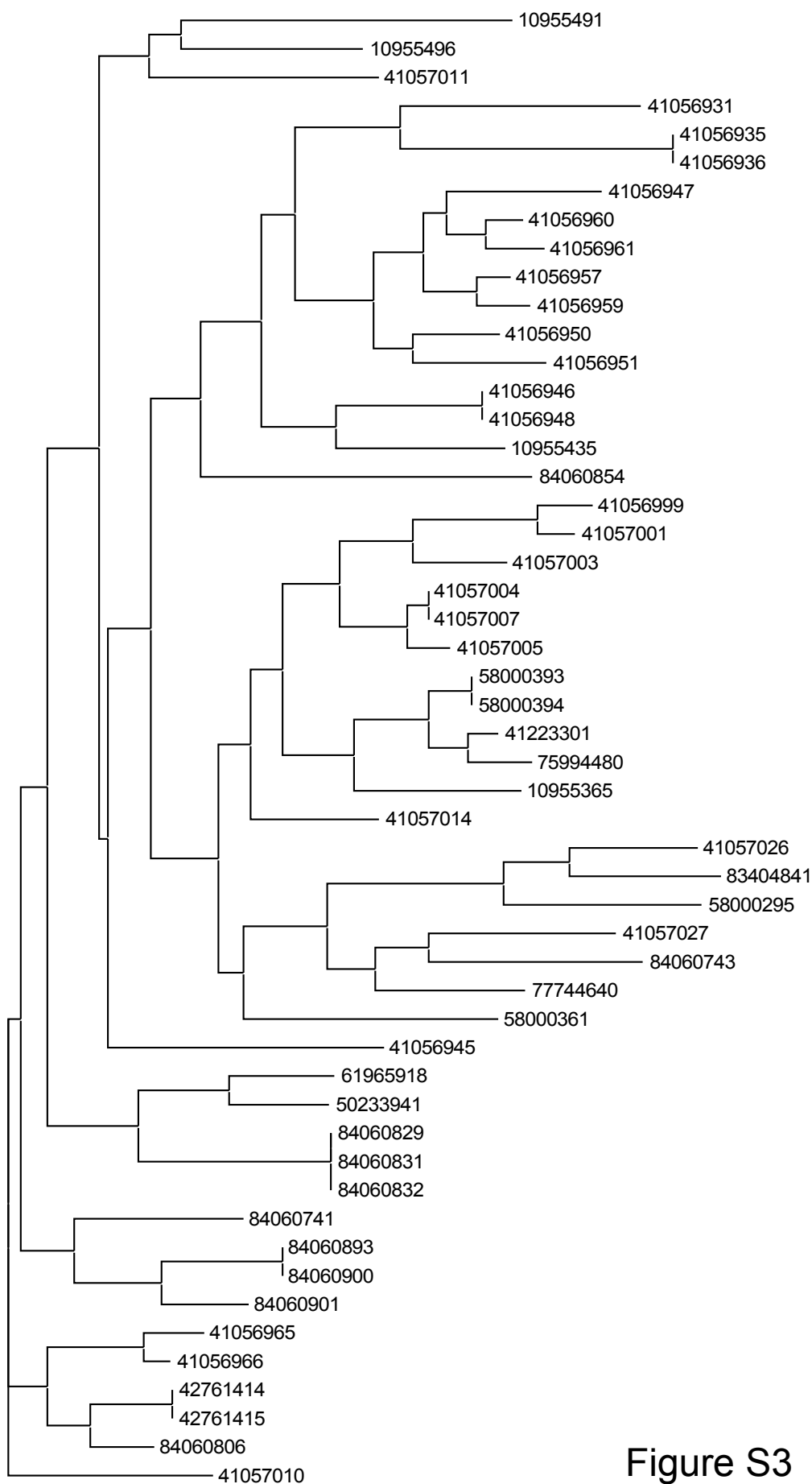

Figure S3

Supplement: Additional file 4 — Figure 3S – Phylogenetic profile with GI numbers of represented proteins as in Figure 5. Protein co-occurrence patterns (see text for details) including the GI numbers of those proteins taken as representatives of each single cluster of Figure 3. [file 1471-2105-9-551-S4.pdf]
